# Burnout and self-reported suboptimal patient care amongst health care workers providing HIV care in Malawi

**DOI:** 10.1371/journal.pone.0192983

**Published:** 2018-02-21

**Authors:** Maria H. Kim, Alick C. Mazenga, Katie Simon, Xiaoying Yu, Saeed Ahmed, Phoebe Nyasulu, Peter N. Kazembe, Stanley Ngoma, Elaine J. Abrams

**Affiliations:** 1 Baylor College of Medicine Children’s Foundation Malawi, Lilongwe, Malawi; 2 Baylor International Pediatric AIDS Initiative at Texas Children’s Hospital, Baylor College of Medicine, Houston, TX, United States of America; 3 Design and Analysis Core, Baylor-UT Houston Center for AIDS Research, Houston, TX, United States of America; 4 Malawi Ministry of Health, HIV Unit, Lilongwe, Malawi; 5 ICAP at Columbia University, Mailman School of Public Health, Columbia University, New York, NY, United States of America; 6 College of Physicians & Surgeons, Columbia University, New York, NY, United States of America; The Ohio State University, UNITED STATES

## Abstract

**Background:**

The well-documented shortages of health care workers (HCWs) in sub-Saharan Africa are further intensified by the increased human resource needs of expanding HIV treatment programs. Burnout is a syndrome of emotional exhaustion (EE), depersonalization (DP), and a sense of low personal accomplishment (PA). HCWs’ burnout can negatively impact the delivery of health services. Our main objective was to examine the prevalence of burnout amongst HCWs in Malawi and explore its relationship to self-reported suboptimal patient care.

**Methods:**

A cross-sectional study among HCWs providing HIV care in 89 facilities, across eight districts in Malawi was conducted. Burnout was measured using the Maslach Burnout Inventory defined as scores in the mid-high range on the EE or DP subscales. Nine questions adapted for this study assessed self-reported suboptimal patient care. Surveys were administered anonymously and included socio-demographic and work-related questions. Validated questionnaires assessed depression and at-risk alcohol use. Chi-square test or two-sample t-test was used to explore associations between variables and self-reported suboptimal patient care. Bivariate analyses identified candidate variables (p < 0.2). Final regression models included variables with significant main effects.

**Results:**

Of 520 HCWs, 62% met criteria for burnout. In the three dimensions of burnout, 55% reported moderate-high EE, 31% moderate-high DP, and 46% low-moderate PA. The majority (89%) reported engaging in suboptimal patient care/attitudes including making mistakes in treatment not due to lack of knowledge/experience (52%), shouting at patients (45%), and not performing diagnostic tests due to a desire to finish quickly (35%). In multivariate analysis, only burnout remained associated with self-reported suboptimal patient care (OR 3.22, [CI 2.11 to 4.90]; p<0.0001).

**Conclusion:**

Burnout was common among HCWs providing HIV care and was associated with self-reported suboptimal patient care practices/attitudes. Research is needed to understand factors that contribute to and protect against burnout and that inform the development of strategies to reduce burnout.

## Introduction

The successful scale-up of antiretroviral treatment (ART) has resulted in an impressive increase in the number of individuals initiating treatment since 2000 [1, 2]. Currently, more than 18 million persons, the majority of whom reside in sub-Saharan Africa (SSA), are receiving ART [1, 2]. Unfortunately, the well-documented shortages of health care workers (HCWs) in SSA are further intensified by the increased human resource needs of rapidly expanding HIV-treatment programs [3–8]. Countries in SSA have 68% of the world's burden of illness from AIDS, yet have only 3% of HCWs worldwide [5]. The combination of staffing shortages and increasing numbers of patients can lead to heavy workloads and workforce burnout, which may result in compromised healthcare outcomes [9–11].

Burnout is a syndrome of emotional exhaustion (EE), depersonalization (DP), and a sense of low personal accomplishment (PA) resulting from chronic job-related stress that can lead to decreased quality of care rendered [12]. The prevalence of burnout among HCWs globally ranges from 15–85%, depending on the medical specialty and working conditions [11, 13–16]. Burnout among HCWs has been shown to be associated with physical and mental illness as well as increased absenteeism, decreased job performance, poor patient practices, and medical errors [10, 17, 18].

Malawi is one of the most resource-limited countries in the world, with one of the lowest per capita GNP in the world at $340 USD [19, 20]. It is a land-locked country of 17.2 million, in Southern Africa. The adult HIV prevalence is high at 10.6%, with 900,000 persons living with HIV/AIDS and an average life expectancy of 64 years [21]. The physician-patient ratio is exceptionally low, with one physician per 62,000 patients, compared to the World Health Organization’s (WHO) recommended ratio of one per 5000 persons [11, 22].

In spite of these limitations in economic and human resources for health, Malawi has implemented an impressive scale-up of HIV treatment. Currently, 679,000 persons are receiving ART, a 2.5-fold increase from 2010 [23, 24]. Moreover, Malawi along with much of Southern Africa has recently launched test-and-treat, an ambitious initiative to offer lifelong ART for all HIV-positive persons upon diagnosis [25, 26]. Although it is a welcome initiative, it threatens to place further strain on an already fragile healthcare system [27]. As resource-constrained countries such as Malawi mount this ambitious ART scale-up, they risk launching an impending crisis of burnout among HCWs that will negatively impact the quality of patient care provided and compromise the tremendous gains in ART expansion. There is a dearth of information from sub-Saharan Africa on burnout among HCWs providing HIV care, especially evidence specifically examining the potential relationship between burnout and quality of patient care. We examined the prevalence and degree of burnout among HCWs providing HIV care in Malawi and explored the relationship of burnout to self-reported patient care practices.

## Methods

### Design

This was a cross-sectional study conducted at 89 public health facilities within 8 districts in the southeastern (districts: Balaka, Machinga, Mangochi, Mulanje, Phalombe, Zomba) and central regions (districts: Lilongwe, Salima) of Malawi from August 2015—January 2016.

### Ethical approval

The National Health Sciences Research Committee in Malawi, as well as the Baylor College of Medicine (BCM) IRB in USA, granted ethical approval.

### Participants and data collection

We approached a convenience sample of HCWs providing clinical care for HIV-positive patients at 89 health facilities in central and southern Malawi to participate in the study. In Malawi, due to the lack of medical doctors, primarily clinical officers, nurses, and medical assistants, all of whom were eligible to participate in the study, provide medical care. Clinicians from Baylor College of Medicine Children’s Foundation Malawi routinely provide bi-monthly clinical mentorship to HCWs with the Ministry of Health. During these routine teaching visits, BCM clinicians approached HCWs providing HIV care and available on the day of the visit to invite them to participate in the study. Of the 539 HCWs available and approached to participate, 535 (99%) consented and enrolled in the study. The BCM clinicians obtained written consent and gave participants the self-administered written surveys. The paper-based surveys were completed anonymously, and all participants were assured that their names would not be written anywhere on the surveys. The survey included the following measures.

### Survey measures

#### Burnout

Burnout was measured using the 22- question Maslach Burnout Inventory (MBI), which has been widely validated and is considered the gold standard measure for burnout [12]. The inventory has been used throughout the world including among HCWs in several countries in Africa and other types of workers in South Africa [6, 11, 28–33]. Each inventory item is rated on a seven-point Likert scale that measures how frequently the respondent experiences a particular feeling (from 0 for *never* to 6 for *everyday*). The MBI measures three constructs of burn out: Emotional Exhaustion (EE), using 9 items to measure physical and emotional depletion; Depersonalization (DP), using 5 items to measure negative or cynical feelings about patients; and Reduced Personal Accomplishment (PA), using 8 items to measure how one perceives one’s own competence. A recent study in Malawi among nurses used the MBI and suggested that it might need modifications to make it more appropriate for use in Malawi (Cronbach alpha scores: 0.67 for EE, 0.42 for DP, and 0.60 for PA) [11]. Informed by this study, we made several modifications to improve clarity and relevance in the Malawi context. The modifications were made with the assistance of Malawian HCWs. The HCWs reviewed the tool and identified questions they found difficult to understand. With study investigators, specific words/statements that were unclear were replaced with clarifying language. For example, for item 1, the term “*drained*” was replaced with *exhausted*; item 11 was modified from “*I worry that this job is hardening me emotionally"* to *"I worry that this job is making me emotionally tough";* and item 20 was modified from "*I feel like I am at the end of my rope" to "I feel like I can't manage at all anymore*.*"* Once changes were made, pretesting was performed among potential participants.

The standardized Cronbach alpha coefficients for the modified MBI were better EE (0.75), DP (0.55), and PA (0.74), as compared to EE (0.67), DP (0.42), and PA (0.60) found in a burnout study previously performed in Malawi [11]. High mean scores on EE and DP scales corresponded to higher degrees of burnout, whereas low scores on the PA subscale corresponded to a high degree of burnout. In line with recent developments in burnout research [9, 17, 34], we chose not to include PA subscales in our overall assessment of burnout, as prior research has suggested this subscale may not belong to burnout syndrome [35, 36].

We defined *burnout* as scores in the mid-high range on the EE (17–54) or DP (7–30) subscales based on cut-off scores used recently among maternal health staff in Malawi [11]. We also calculated a global burnout score to examine the relationship between burnout as a unified entity and its potential for dose-response relationship with suboptimal patient care. To calculate the global burnout score we used the weighting system developed by Bianchi et al (0.6 emotional exhaustion + 0.4 depersonalization) for their study on burnout [34, 37]. This weighting scheme recognizes the primacy of emotional exhaustion in burnout [38, 39].

#### Self-reported patient care practices and attitudes

We measured suboptimal patient care practices and attitudes with nine statements adapted from prior research that investigated self-reported patient care amongst health care workers [17, 40]. A group of health care workers providing HIV care in Malawi modified the statements to present HIV-focused patient care practices that are common, relevant and important to provide HIV care. These statements were: (1) I did not welcome the patient politely; (2) I did not fully discuss the treatment plan or answer a patient’s questions; (3) I made mistakes in treatment or medication that were not due to a lack of knowledge/inexperience; (4) I did not perform a diagnostic test because of desire to finish up with the patient quickly; (5) I have sometimes been absent from work when I was not supposed to be; (6) I shouted at a patient; (7) I paid little attention to how much HIV might affect a patient personally or socially; (8) I had little emotional reaction to the death of one of my patients; (9) I felt guilty about how I treated a patient from a humanitarian standpoint.

The questionnaire did not explicitly use the word *suboptimal* or suggest that these practices/attitudes were undesirable. Participants were asked to rate how frequently (never, few times a year or less, once a month or less, few times a month, weekly, few times a week or every day) they participated in certain behaviors using the prior year as reference. We created a summary measure for self-reported suboptimal patient-care practices defined as one or more suboptimal patient-care practices reported more frequently than monthly to a few times a month, hereafter called *suboptimal patient care monthly*. This summary outcome measure was used for the univariate and multivariate regression analyses. Because of the similarity of questions related to the DP component of the MBI and suboptimal patient-care attitudes, as in prior studies [17, 40], we excluded suboptimal patient-care attitudes from the summary outcome measure.

#### Depression and substance abuse

The World Health Organization’s (WHO) self-reporting questionnaire (SRQ) is a widely validated screening tool for depression [41]. It has been validated in Malawi and was used in this study [42]. Based on cut-off scores used in other studies, including those done in Malawi, a cut-off score of 8 was defined as a positive screen for depression. Depression was also evaluated as a continuous variable. We also asked about prior history of depression. The standardized Cronbach’s alpha for the SRQ in our study was an acceptable 0.79, similar to prior work done in Malawi using the SRQ [42] (Cronbach's alpha, 0.85).

The widely validated WHO Alcohol Use Disorders Identification Test (AUDIT) [43] was used to screen for at-risk use of alcohol. A total score is calculated by summing individual item responses across all 10 items. A higher score indicates more problematic use of alcohol. As per AUDIT instructions [43], items 9 and 10 were scored such that “No” = 0; “Yes, but not in the past year” = 2; and “Yes, during the past year” = 4. Based on cut-off scores recommended by WHO and research from studies in Malawi and South Africa, a total score of 8 was considered a positive screen for potentially hazardous or harmful use of alcohol [43–45]. To measure use of other recreational drugs, we asked “How often do you use other drugs (marijuana, etc.),” and the score was adapted from the DUDIT [46].

#### Demographic, patient and work characteristics

Participants’ self-reported demographics and work characteristics included continuous variables: age, number of children, years since completing training and number of years worked as a HCW; binary variables: gender, HCW cadre and provision of HIV care; and other categorical variables: type of health facility, time spent providing direct clinical care and number of hours worked in a typical week.

### Data analysis

Data were summarized by descriptive statistics (mean, SD, median, IQR, frequency). Chi-square test, two-sample t-test, and Fisher’s exact test were used to explore the associations between potential factors and sub-optimal patient care practiced monthly.

Logistic regression models were used to examine the association between burnout and self-reported suboptimal patient care, while controlling for other variables. We performed univariate screening by bivariate analysis. Variables were selected for inclusion in the model selection if their p-value was < 0.20. A backwards selection procedure was applied with a significance level of 0.05. Only variables with a p-value < 0.05 were retained in the final model. The scale for continuous variables was examined using quartiles to ensure a linear assumption was met prior to entry into the logistic model. We used 95% confidence intervals for the estimates.

Missing items on the MBI were imputed using mean substitution for the same subscale and the same participant [37, 47, 48]. We also conducted sensitivity analysis, by analyzing data using subjects with all completed items. All results were very similar. All analyses were performed using SAS software version 9.4 (SAS Institute, Inc., North Carolina, USA).

## Results

A total of 535 HCWs enrolled in the study (Table 1). Of these, 15 completed surveys were excluded due to significant missing data. The mean age (SD) was 34 (10.2) years, 59% were female, 58% were married, and 37% were medical officers, clinical officers, or medical assistants. The majority (88%) provided clinical care more than 75% of the time, 36% worked more than 60 hours a week, 7% had a positive depression screen, 5% reported a history of depression, 6% had at risk use of alcohol, and only 4 participants reported using drugs.

**Table 1:** Characteristics of participants. N = 520.

**Age**, years, mean (SD)	34 (10.2)
**Gender,** female, n (%)	305 (59)
**Marital Status**, n (%)	
Married	301 (58)
Widowed/divorced	40 (8)
Single	178 (34)
Missing	1
**No of children**, median (IQR)	1 (0–2.0)
Missing	6
**Children less than 5 years old**	
No	327 (64)
Yes	187 (36)
Missing	6
**Type of HCW**, n (%)	
Medical Officer/Clinical officer/Medical assistant	190 (37)
Nurse midwife technician/state registered nurse	330 (63)
**Length of time from completing health care worker training**, years, median (IQR)	6.0 (3.0–10.0)
Missing	2
**Years worked as a health care worker**, median (IQR)	5.0 (3.0–10.0)
Missing	1
**Provided clinical care to persons living with HIV in the past month**, n (%)	420 (81)
**Health facility type**, n (%)	
District Hospital	134 (26)
Rural Hospital	90 (17)
Health center or other	296 (57)
**Number of hours worked in a week**, n (%)	
Less than 40 hours	35 (7)
40–50 hours	230 (44)
51–60 hours	68 (13)
More than 60 hours	184 (36)
Missing	3
**Time spent providing direct clinical care**, n (%)	
All of my time	249 (48)
>75%	205 (40)
50%	41 (8)
<50% or don’t provide clinical care	21 (4)
Missing	4
At-risk alcohol use* n (%)	33 (6)
**Depression- positive screen**^#^, n (%)	36 (7)
Missing	1
**Suicidal Ideation**, n (%)	20 (4)
Missing	1

*As measured by AUDIT (Alcohol Use Disorders Identification Test) score >8

#World Health Organization, Self-Reporting Questionnaire (SRQ), cut off 8

Table 2 summarizes burnout, MBI subscale scores, and self-reported patient care practices and attitudes. For the MBI, 98.3% of participants responded to all items; 9 participants had 1 item missing, 1 participant had 2 items missing, and 1 participant had three items missing. No participants were missing more than 1 item in one subscale. The majority (62%) of HCWs met criteria for burnout. In the three dimensions of burnout, 55% reported moderate-high EE, 31% moderate-high DP, and 46% low-moderate PA. The vast majority of HCWs (89%) reported having at least one suboptimal patient care practice or attitude more than a few times a year or less, 66% reported more than once a month or less, and 41% reported at least weekly. Suboptimal patient care practices were reported by 50% of participants. Self-reported suboptimal care included making mistakes in treatment not due to lack of knowledge/experience (52%), not fully discussing the treatment plan or answering patient questions (52%), shouting at patients (45%), not welcoming the patient politely (43%), not performing diagnostic tests due to a desire to finish up quickly (35%), and absenteeism (24%).

**Table 2:** Burnout out and self-reported suboptimal patient care practices and attitudes, n = 520.

**Burnout**	
Mod-high emotional exhaustion (EE) or mod-high depersonalization (DP), n (%)	321 (62)
Mod-high EE or mod-high DP or low-mod PA, n (%)	405 (78)
Global burnout score^#^, mean (SD)	13.0 (7.3)
**Maslach Burnout Index subscales**	
Moderate to High score for EE, n (%)	284 (55)
Mean (SD), and Median (IQR)	18.3 (10.3), 18 (10–25)
Moderate to High score for DP, n (%)	161 (31)
Mean (SD), and Median (IQR)	5.0 (5.0), 4 (1–7)
Low to Moderate score for PA, n (%)	237 (46)
Mean (SD), and Median (IQR)	37.6 (9.2), 40 (33–44.5)
**Self-reported suboptimal patient care/attitudes practiced, n (%)**	
more than a few times a year or less	463 (89)
more than once a month or less	342 (66)
a few times a month or less	271 (52)
at least weekly	214 (41)
**Self-reported suboptimal patient care practiced, n (%)**	
more than a few times a year or less	416 (80)
more than once a month or less	259 (50)
a few times a month or less	173 (33)
at least weekly	108 (21)
**Self-reported suboptimal patient care practiced a few times a year or less n (%)**	
I did not welcome the patient politely	222 (43)
I did not fully discuss the treatment plan or answer a patient’s questions	271 (52)
I made mistakes in treatment or medication that were not due to a lack of knowledge/inexperience	268 (52)
I did not perform a diagnostic test because of desire to finish up with the patient quickly	181 (35)
I have sometimes been absent from work when I was not supposed to be	127 (24)
I shouted at a patient	232 (45)
**Self-reported suboptimal patient care attitudes a few times a year or less n(%)**	
I paid little attention to how much HIV might affect a patient personally or socially	158 (30)
I had little emotional reaction to the death of one of my patients	226 (43)
I felt guilty about how I treated a patient from a humanitarian standpoint	363 (70)

# Global burn out score = [0.6*EE + 0.4*DP]

EE (emotional exhaustion) subscale cut offs: High ≥27, Moderate 17–26. DP (depersonalization) subscale cut offs: High ≥13, Moderate 7–12. PA (personal accomplishment) subscale cut offs: Low ≤31, Moderate 32–38. Moderate to high score for EE was 17–54, moderate to high score for DP was 7 to 30, low to moderate score for PA was 0 to 38.

Table 3 displays results of univariate analyses examining the associations between self-reported suboptimal patient care and other variables. The only variable found to be significantly associated with self-reported suboptimal patient care (p<0.05) was burnout. HCWs who met criteria for burnout were more likely to report suboptimal patient care (42.4% vs. 18.6%, p<0.0001). The EE and DP subscales (p <0.001), but not the PA subscale (p = 0.18), were found to be associated with providing suboptimal patient care.

**Table 3:** Factors associated with self-reported suboptimal patient care practices- more than once a month–univariate analysis.

Variable	Suboptimal care No	Suboptimal care Yes	p-value
**Age**, years, mean (SD)	33.5 (9.8)	34.34 (11.0)	0.38*
**Gender,** n (%)			0.40
Male	139 (64.7)	76 (35.3)	
Female	208 (68.2)	97 (31.8)	
**Marital Status**, n (%)			0.72
Married	205 (68.1)	96 (31.9)	
Widowed/divorced	25 (62.5)	15 (37.5)	
Single	117 (65.7)	61 (34.3)	
**No of children**, median (IQR)	1 (0–2)	1 (0–3)	0.94*
**Children less than 5 years old**			0.374
No	213 (65.1)	114 (34.9)	
Yes	129 (69)	58 (31)	
**Type of HCW**, n (%)			0.09
Medical Officer/Clinical officer/Medical assistant	118 (62.1)	72 (37.9)	
Nurse midwife technician/state registered nurse	229 (69.4)	101 (30.6)	
**Length of time from completing health care worker training**, years, median (IQR)	8.5 (9.0)	9.1 (9.9)	0.50*
**Years worked as a health care worker**, median (IQR)	8.7 (8.9)	9.2 (9.8)	0.55*
**Provided clinical care to persons living with HIV in the past month**, n (%)			0.48
No	69 (69.7)	30 (30.3)	
Yes	277 (66)	143 (34)	
**Health facility type**, n (%)			0.07
District Hospital	99 (73.9)	35 (26.1)	
Rural Hospital	62 (68.9)	28 (31.1)	
Health center or other	186 (62.8)	110 (37.2)	
**Number of hours worked in a week**, n (%)			0.67
Less than 40 hours	24 (68.6)	11 (31.4)	
40–50 hours	159 (69.1)	71 (30.9)	
51–60 hours	43 (63.2)	25 (36.8)	
More than 60 hours	18 (64.1)	66 (35.9)	
**Time spent providing direct clinical care**, n (%)			0.42
All of my time	172 (69.1)	77 (30.9)	
>75%	134 (65.4)	71 (34.6)	
50%	23 (56.1)	18 (43.9)	
<50% or don’t provide clinical care	14 (66.7)	7 (33.3)	
At-risk alcohol use* n (%)			0.06
No	330 (67.8)	157 (32.2)	
Yes	17 (51.5)	16 (48.5)	
**Depression- positive screen**^**#**^, n (%)			0.07
No	327 (67.7)	156 (32.3)	
Yes	19 (52.8)	17 (47.2)	
**Suicidal Ideation**, n (%)			0.11
No	337 (67.5)	162 (32.5)	
Yes	10 (50)	10 (50)	
**History of Depression**, n (%)			0.37
No	333 (67.1)	163 (32.9)	
Yes	14 (58.3)	10 (41.7)	
**Burnout: MBI, mod-high EE or mod-high DP**			<0.0001
No	162 (81.4)	37 (18.6)	
Yes	185 (57.6)	136 (42.4)	
**Burnout: global score**, mean (SD)	11.47 (6.86)	16.15 (7.27)	<0.0001*

Analyses by chi-square test unless otherwise noted.

* Two-sample t-test

Five variables (cadre of HCW, health facility type, burnout, depression- positive screen and at-risk alcohol use) were included in the multivariate logistic regression model. Burnout was the only variable that remained associated with suboptimal patient care (EE+DP, odds ratio [OR] 3.22, [CI 2.11 to 4.90]; p<0.0001). When the EE and DP dimensions were examined separately, they both remained independently associated with suboptimal patient care: (EE, OR 2.03, [CI 1.34 to 3.06]; p = 0.0008) and (DP, OR 3.20, [CI 2.12 to 4.82]; p<0.0001).

To determine if a dose-response relationship existed between burnout and suboptimal patient care practices monthly, we used the global burnout score. With each unit increase in the global burnout score, there was a 1.10-fold change in the odds of providing suboptimal patient care monthly (OR 1.10, [CI 1.07 to 1.13]; p<0.0001).

**Fig 1:**
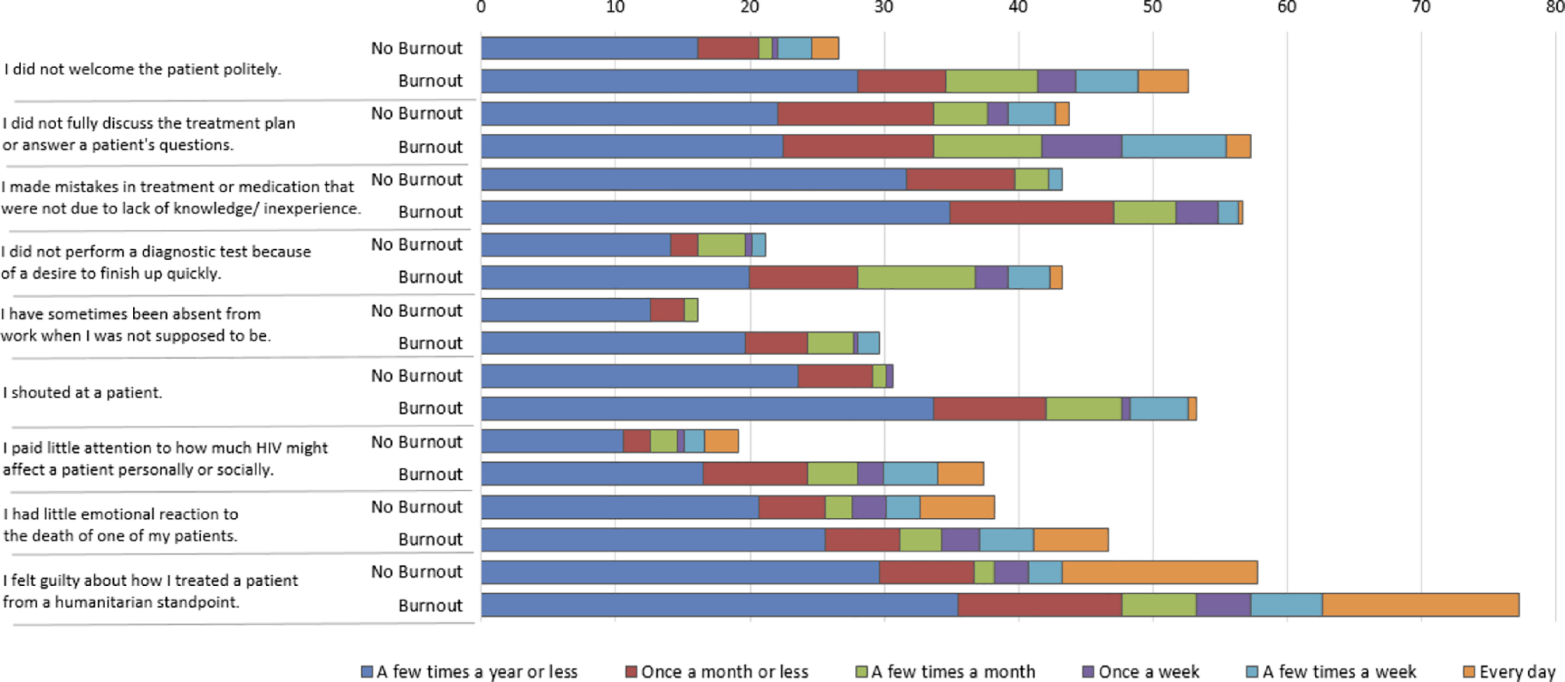
Relationship of burnout (moderate/high EE or moderate/high DP) to self-reported suboptimal patient care practices and attitudes.

Fig 1 illustrates a comparison of self-reported rates of various suboptimal patient care practices and attitudes between HCWs who met criteria for burnout versus those who did not. The top bar represents HCWs who did not meet criteria for burnout. The bottom bar represents HCWs who met criteria for burnout. More HCWs who met criteria for burnout self-reported each of the nine suboptimal patient care practices/attitudes.

## Discussion

To our knowledge, this is one of the largest published studies of HCW burnout in SSA and among the first to report a relationship between HCW burnout and patient care. Burnout was commonly experienced among the HCWs in our study: more than 60% met criteria for burnout. A disconcertingly high proportion of HCWs, almost 90%, self-reported engaging in suboptimal patient care practices or attitudes, 66% reported doing it monthly and 41% weekly. HCWs who were burned out were more likely to report providing suboptimal patient care monthly. Although we are unable to determine causality due to time-varying confounding, we observed that burnout was associated with a 3.2 times increased odds of reporting a suboptimal patient care practice. Each one-point increase in global burnout score was associated with a 10% higher likelihood of reporting suboptimal patient care.

Burnout is defined as when HCWs feel emotionally exhausted, cynical and detached, and ineffective and when they feel “an erosion in values, dignity, spirit, and will” [49, 50]. HCWs are the backbone of an optimally functioning health system, so when HCWs are not well, the performance of the health care system suffers [49, 50].

Unfortunately, HCWs in SSA often work under burnout-inducing conditions: long hours, high and increasing burden of responsibility, low perceived control, unsupportive environments, staffing shortages, and the crushing weight of high rates of patient morbidity and mortality [51–53]. Nonetheless, there has been a dearth of research on and attention given to HCW burnout in sub Saharan Africa (SSA).

In comparison, research on burnout in developed countries is well established, and the field has now largely moved towards examining potential interventions. In a recent comprehensive meta-analysis on burnout prevention and treatment interventions [54] only three of 52 studies included were done in low-income countries.

Why has burnout in SSA received comparatively little attention? In the West, when burnout was seen as a crisis of well being among privileged physicians, it elicited little public sympathy. However, as mounting evidence began to suggest that burnout negatively affects physicians’ effectiveness and patient safety, health planners and the public became justifiably worried about the impact of burnout on the quality of patient care [50]. Perhaps similarly in SSA, set against the backdrop of pressing and unmet patient needs, HCW burnout, when viewed as affecting only the HCWs themselves, has not been deemed a priority. More evidence regarding how significant the problem of burnout might be, as well as how burnout might negatively affect health services output in SSA, may help health planners acknowledge human limitations when layering increasing demands on HCWs [50].

More than half of the participating HCWs met criteria for burnout, with 55% reporting moderate-high EE, 31% moderate-high DP, and 46% low-moderate PA. These findings were similar to those found in other studies among HCWs in Malawi (66% moderate-high EE, DP moderate-high 69%) [55], two studies in Zambia (in one study, 51% were defined as burned out [3], and in the other study 62% felt moderate-to-high EE) [6], a study amongst nurses in Nigeria [28] (39% reported moderate-to-high EE), voluntary medical male circumcision providers (Kenya (66%), South Africa (33%), Zimbabwe (17%), and Tanzania (15%) reported starting to experience work fatigue/burnout) [56]. MBI cut-off points can vary by country based on socio-cultural reasons [57], and, therefore, comparisons should be interpreted with this limitation in mind. However, the rates of burnout are similar to those found in other studies among HCWs in the region, and the high proportion of HCWs meeting burnout criteria is concerning.

An alarming majority of HCWs, almost 90%, self-reported suboptimal patient care practices and attitudes; 80% reported suboptimal patient care practices, with 50% reporting it monthly. Suboptimal patient care practices included admitting mistakes in treatment not due to lack of knowledge/experience (52%), shouting at patients (45%), not performing diagnostic tests due to a desire to finish quickly (35%), and absenteeism (24%). Provider-patient relationships have been shown repeatedly to impact patient outcomes. Disrespectful treatment can lead to patients not engaging in or abandoning treatment, whereas healthy relationships can enhance HIV care [58–65]. Despite this evidence, as well as the significant investment in the improvement of health systems in SSA, there is a surprising dearth of evidence regarding the quality of HCW patient care practices in SSA, and these results highlight the urgent need for additional examination.

We found that burnout was the only variable that remained associated with suboptimal patient care. HCWs who met criteria for burnout had a 3.2 times increased odds of reporting a suboptimal patient care event. Each one-point increase in a global burnout score was associated with a 10% higher likelihood of reporting a suboptimal patient-care event. Our findings are consistent with preliminary research on burnout from resource-limited settings that have shown that HCW burnout is associated with negative effects on health care delivery [10, 17, 40]. Further, it is possible that the reports from SSA on the increasing challenges with HCWs’ absenteeism [66], negative treatment of patients, and high turnover [67], may result in part from HCWs’ burnout. We hope that our observation regarding the relationship between burnout and self-reported suboptimal boosts enthusiasm for additional research and action.

This study has several limitations. We used a convenience sample of HCWs who were available during routine program visits. The definition and reporting of suboptimal patient care were based on self-report. However, other studies have also used self-report as a measure of suboptimal patient care [17, 40, 68]. Due to time-varying confounding we cannot determine the causal direction of the association found between burnout and suboptimal patient care. In addition, there are likely unmeasured confounders. For example, certain personality disorders (cynicism, narcissism, high negativity-personality) may have influenced individuals’ perceptions of patient-care practices and could be examined in future work. We did measure and adjust for several important potential confounders such as depression, alcohol use, and work hours. However, the current study cannot account for more complex mechanisms such as time-varying confounding. Despite these limitations, to our knowledge, there are no published studies from SSA that examine the relationship between burnout and patient care, and, therefore, our findings help address a clear gap.

Strengths of the study include a large sample size both in terms of the number of HCWs who participated and the number of representative healthcare facilities (>89 facilities). The response rate was very good, and the surveys were conducted anonymously to reduce social-desirability bias. Another strength is that in line with recent developments describing that PA likely does not fall under burnout syndrome, we excluded PA in our burnout criteria. Finally, this study was conducted in Malawi, where the HCW workforce is already strained and expectations are increasing with the test and treat all approach and integration of outpatient treatment for non-communicable diseases [27, 69].

The HCWs who participated in this study, like many HCWs throughout SSA, not only provide HIV care but are the critical backbone of health care systems, functioning as the frontline providers for most outpatient health services. They are very likely the same HCWs who are tasked to attend to the increasing patient load from the test-and-treat approach and will be called upon to address the rising need to address care of non-communicable diseases in developing countries. Even before the inauguration of the ambitious test-and-treat approach, research on ART scale-up highlighted the importance of limiting burnout and attrition to achieve a program’s maximum efficiency [70]. Now, through the great success of ART scale-up, we are faced with clear concerns around long-term retention of patients on ART, which is where HCWs’ attitudes may have the greatest influence. The additional strain on an under-resourced healthcare system may exacerbate the already significant levels of burnout.

Burnout among HCWs providing HIV care is a critical and impending challenge that requires urgent attention to prevent compromising treatment gains. Additional research is needed to better characterize burnout in SSA, understand its relationship to patient outcomes, and inform the development of strategies to reduce burnout.

## Supporting information

S1 File Maslach- adapted. (PDF)
