# Supplementary material for: Burnout and self-reported suboptimal patient care amongst health care workers providing HIV care in Malawi
Source: PLoS One. 2018 Feb 21;13(2):e0192983. doi: 10.1371/journal.pone.0192983 (PMC5821338; doi:10.1371/journal.pone.0192983)
Supplement: S1 File — (PDF) [file pone.0192983.s001.pdf]

MASLACH ADAPTED July 2015

Baylor College of Medicine Children's Foundation Malawi, Tingathe Programme

The purpose of this survey is to discover how various persons In the human services, or helping professionals view their job and the people with whom they work closely.

Because persons in a wide variety of occupations will answer this survey, it uses the term *recipients* to refer to the people for whom you provide your service, care, treatment, or instruction. When answering this survey please think of these people as recipients of the service you provide, even though you may use another term in your work.

Instructions: On the following pages are statements of job-related feelings. Please read each statement carefully and decide if you ever feel this way about *your* job. If you have *never* had this feeling, write the number "0" (zero) in the space before the statement. If you have had this feeling, indicate *how often* you feel it by writing the number (from 1 to 6) that best describes how frequently you feel that way. An example is shown below.

Example:

**How Often:**	**0** Never	**1** A few times a year or less	**2** Once a month or less	**3** A few times a month	**4** Once a week	**5** A few times a week	**6** Every day

	**How Often 0-6**	**Statement:**
1.	__________	I feel depressed at work.

If you never feel depressed at work, you would write the number "0" (zero) under the heading "How Often." If you rarely feel depressed at work (a few times a year or less), you would write the number "1." If your feelings of depression are fairly frequent (a few times a week but not daily), you would write the number "5."

MASLACH ADAPTED July 2015

Baylor College of Medicine Children's Foundation Malawi, Tingathe Programme

Part A:

MBI-Human Services Survey

**How Often**	**0**<br>Never	**1**<br>A few times a year or less	**2**<br>Once a month or less	**3**<br>A few times a month	**4**<br>Once a week	**5**<br>A few times a week	**6**<br>Every day

**How Often 0-6**	**Statements:**
1.__________	I feel emotionally exhausted from my work.
2. _________	I feel used up at the end of the workday.
3. _________	I feel fatigued when I get up in the morning and have to face another day on the job.
4. _________	I can easily understand how my patients feel about things.
5. _________	I feel I treat some patients as if they were impersonal objects.
6. _________	Working with people all day is really a strain for me.
7. _________	I deal very effectively with the problems of my patients.
8. _________	I feel burned out from my work.
9. _________	I feel I'm positively influencing other people's lives through my work.
10. _________	I've become more unsympathetic toward people since I took this job.
11. _________	I worry that this job is hardening me emotionally.
12. _________	I feel very energetic.
13. _________	I feel frustrated by my job.
14. _________	I feel I'm working too hard on my job.
15. _________	I don't really care what happens to some patients.
16. _________	Working with people directly puts too much stress on me.
17. _________	I can easily create a relaxed atmosphere with my patients.
18. _________	I feel energized after working closely with my patients.
19. _________	I have accomplished many worthwhile things in this job.
20. _________	I feel like I don't have energy to cope with my work anymore.
21. _________	In my work, I deal with emotional problems very calmly.
22. _________	I feel patients blame me for some of their problems.
